# The positive side of a negative reference: the delay between linguistic processing and common ground

**DOI:** 10.1098/rsos.160827

**Published:** 2017-02-08

**Authors:** Edmundo Kronmüller, Ira Noveck, Natalia Rivera, Francisco Jaume-Guazzini, Dale Barr

**Affiliations:** 1 Escuela de Psicología, Pontificia Universidad Católica de Chile, Santiago, Chile; 2 Laboratoire sur le Langage, le Cerveau et la Cognition (UMR5230), France; 3 Amsterdam Center for Language and Communication, Universiteit van Amsterdam, The Netherlands; 4 Escuela de Educación, Universidad Mayor, Santiago, Chile; 5 Department of Psychology, University of Glasgow, Glasgow, UK

**Keywords:** dialogue, language, common ground, reference, negation

## Abstract

Interlocutors converge on names to refer to entities. For example, a speaker might refer to a novel looking object as *the jellyfish* and, once identified, the listener will too. The hypothesized mechanism behind such *referential precedents* is a subject of debate. The common ground view claims that listeners register the object as well as the identity of the speaker who coined the label. The linguistic view claims that, once established, precedents are treated by listeners like any other linguistic unit, i.e. without needing to keep track of the speaker. To test predictions from each account, we used visual-world eyetracking, which allows observations in real time, during a standard referential communication task. Participants had to select objects based on instructions from two speakers. In the critical condition, listeners sought an object with a negative reference such as *not the jellyfish*. We aimed to determine the extent to which listeners rely on the linguistic input, common ground or both. We found that initial interpretations were based on linguistic processing only and that common ground considerations do emerge but only after 1000 ms. Our findings support the idea that—at least temporally—linguistic processing can be isolated from common ground.

## 1. Introduction

When a mother glances at her son, says ‘Look’ and points ahead, she expects her son to understand that her intention was to guide his sight to, let us say, a spectacular full moon on the horizon; likewise, the son needs to work out that the mother's intention was for him to notice the moon. Reference resolution is arguably one of the most basic of pragmatic acts and so referential phenomena fit well within an ostensive-inferential framework of communication as first conceived by Paul Grice [1], according to which the linguistically encoded meaning of an utterance is just part of the listener's effort to understand the speaker's intention.

Reference resolution comes in various forms. When it involves pointing, the observer needs to distinguish the indicated object from countless possibilities and understand precisely what is the pointer's purpose [2]. When identifying an object, e.g. as *the small cup*, the speaker is also potentially providing a listener with a means to distinguish that object from other similar ones, i.e. non-small cups [3,4]. When a speaker labels an atypical object as *the jellyfish* it is a way for him to have common vocabulary with his listener [5,6]. What ties these examples together is that all of them call on some amount of intention reading. The related question in the experimental literature is, how much is *common ground*, which comprises the set of beliefs, assumptions and suppositions that interlocutors share and know that they share [7], implicated in reference resolution?

In what follows, we will describe in greater detail one of the referential phenomena described above—precedent uses (as in the example above concerning *the jellyfish*). We will then present two opposing points of view about the way it works. Briefly, one school of thought, starting with Clark [8], is that a listener should comprehensively and automatically incorporate features of common ground when resolving reference. Another school, starting with Keysar and co-workers [9,10], defends a minimalist position which says that the referential utterance may be a starting point for listeners to access features of common ground but it may not even be called upon nor be necessary. The intense exchange between the two sides has allowed researchers to sharpen their collective vision about the way referential resolution is carried out. We then turn to our experiment which aims to determine—in a finely tuned eye-tracking paradigm—the point at which intentional factors, such as common ground, come into play in the resolution of re-used referents.

### 1.1. Precedents and speaker consistency

When speakers introduce a referent into discourse, their description establishes a referential precedent that interlocutors tend to follow subsequently [5,6]. For example, Brennan & Clark [11] showed that speakers who have referred to a particular couch as *the old couch* tend to continue to refer to it that way, even when a change in the context makes the description overly specific (e.g. when it becomes the only couch in question). Maintaining consistency helps interlocutors assign reference and to such an extent that it often overrides the goal of providing listeners with optimally informative descriptions in the immediate context. This consistency arguably reduces ambiguity in conversation and is found even among 3–5-year-old children [12,13]. A wide range of studies has now shown that listeners are able to exploit this collaborative consistency [9,14–19].

Despite consensus around the idea that comprehension is guided by referential precedents, there has been considerable debate over the nature of this guidance. According to Brennan and colleagues, referring precedents reflect *conceptual pacts*, which are mutually accepted agreements between specific interlocutors [11,17]. When a speaker chooses a particular way of conceptualizing and describing a referent, she does it in a way that facilitates a listener's interpretation; and when a listener (tacitly) accepts this description, the link between the conceptualization and the referent becomes part of their common ground. Thus, the influence of referential precedents on interpretation arises from two key aspects of a dialogue: the dynamics of collaboration, through which interlocutors strive to mutually facilitate comprehension by making their contribution as easy as possible to interpret, and common ground, a set of shared assumptions or shared knowledge that arises through joint actions [20,21].

This view predicts that precedents established between the listener and only one interlocutor ought to influence the listener when identifying referents, either completely or partially [14,17]. This means that efficient reference resolution will directly depend on the identity of the speaker. This has led to two predictions. The first is that a speaker's identity and common ground ought to matter when a speaker, in a one-on-one conversation, employs a second expression to describe a previously named object. Concretely, a speaker who changes the name of a previously named referent ought to prompt consternation from a listener; meanwhile, a newly arriving speaker who comes up with a new name for an object that was previously named by another interlocutor ought not to surprise the listener. For example, if a speaker A named a novel-looking object as ‘the upside-down funnel’, the listener should be surprised if that speaker does not re-use the expression to refer to it again, using instead ‘the black vase’. This surprise should not happen if a new speaker (B) uses that new expression to refer to the same object. The second, related prediction is that a listener should benefit—mainly by facilitating reference resolution—from a speaker when she re-uses an expression to refer to an object; moreover, this benefit should be less if a second interlocutor, who is naive to a pair's prior conversation, were to coincidentally refer to a previously named object by using the same expression that was established by the original pair.

In an influential paper, Metzing & Brennan [17] tested the first prediction by tracking participants’ eye movements as they followed directions from a confederate speaker in order to select one object among several. The authors found that when the speaker uses a new expression to identify a previously named item, i.e. the intended object, participants take longer to select it, even if it is the one that best fits the description. Importantly, this delay only occurs when the previous name was a precedent in common ground with that speaker. That is, when a new, naive interlocutor applies that new expression to identify a previously named item (the one that had been assigned a name through common ground with another speaker), there is no apparent delay in selecting the object that best fits the description.

Recent work, however, questions both claims derived from this view. With respect to the first prediction, Kronmüller & Barr [19] conducted a meta-analysis of 10 experiments that used a similar paradigm and found that listeners do momentarily look away from an object that already has a name attached to it, no matter who did the naming. Experimental work does not support entirely the second prediction either. Established precedents that are repeated by someone other than the initial speaker appear to be understood as readily as those that come from the one who originally coined the referent [9,17]; there is a short-lived facilitation with the original speaker, but that facilitation fades away quickly after its appearance [14,22]. A new speaker could use a previously used expression and the listener would hardly notice nor will it have a strong influence on comprehension. Therefore, there is evidence that any available precedent that listeners establish with a previous speaker will influence performance in a reference-naming task. In their meta-analysis, Kronmüller and Barr also found that the comprehension of precedents is largely speaker-independent; that is, reference-comprehension is not steadfastly linked to shared information between a speaker and a listener.

This leads to an alternative view according to which re-used expressions are best viewed as linguistic expressions that have no immediate recourse to the identity of the speaker and, it follows, to common ground. This explanation is in line with accounts of egocentrism that have been documented widely in the literature [10,23], according to which interlocutors are harboured to their own perspective and do not readily consider their interlocutor's (also see Hanna *et al.* [24]). For example, when a participant sees three differently sized candles with only the smallest outside the speaker's purview and then hears a speaker say ‘take the small candle and …’, the participant usually looks at or even moves the smallest one anyway. The experimental case of precedent use indicates that the extraction of a more comprehensive intentionally rich meaning, one that incorporates concerns about common ground, is not automatic. This is consistent with the account by Grice [1], who pointed to a gap between the processing of linguistic meaning and a fully formed intentional one.

### 1.2. Our approach: the current study

We propose that a referential precedent, even a newly coined one, can be viewed practically as a linguistic unit. Like in any utterance, linguistic meanings have a role to play in a listener's effort to work out the speaker's intention. It follows that features like common ground have an important role to play, too. However, this should not be taken to mean that participants in referential paradigms always need to fully consider pragmatic features, such as speaker identity and common ground. The question we pursue here is, when does common ground come into play? The strong position would argue that common ground's role is immediate and that common ground features (concerning the speaker's identity) are part and parcel of referential expression [14,21,25]. According to our approach, linguistic expressions are the basis for eventually working out the speaker's intention. This leads to two possible outcomes. Either participants stick with the linguistic meaning and fail to fully engage common ground features or they process the linguistic meaning and eventually bring common ground features into consideration. Either way, one should see evidence showing that a reading based on an utterance's linguistic meaning can be isolated from a reading that takes full advantage of its pragmatic potential.

To create this diagnostic study, we adapted the now classic paradigm known as Referential Communication Game [5,26], in which novel-looking objects are given original names by a speaker (known as a Director in the prior studies). Participants see several novel-looking objects on a computer screen and are asked by a live confederate, for example, to ‘click on (the one that looks like) the jellyfish’. To introduce a new speaker into the scenario, another interlocutor—who is off-stage, as well as unknown to the confederate, and whose voice was previously recorded—intervenes (through headphones) from time to time to locate objects on the screen with his own original names as well. This much conforms to prior studies. Meanwhile, participants’ eye movements are recorded, in the context of the Visual World Paradigm, in order to reveal real-time language processing (for a review of this paradigm, see Huettig *et al.* [27]). The innovation here is twofold. The first concerns participants’ beliefs about the situation: participants are led to believe that the interlocutor is viewing just two objects while the participant is viewing three. Therefore, one of the three objects on the participant's display is an ‘extra’ object that is not seen by the speaker; moreover, the listener does not know in advance which object it is. Additionally, the speaker thinks that the participant sees the same two objects he sees. This becomes important when the participant has to reason through the speaker's critical test sentence.

The second innovation is that the critical test sentence uses negation. We introduced negation because, while its basic meaning is to deny propositions, it can lead a listener to carry out a search for alternatives [28] (for a discussion, see Noveck [29]). As Oaksford [30] has argued, negations prompt a listener to construct a contrast class. In the current experiment, it is our expectation that negative referential expressions such as ‘not the jellyfish’ will lead the listener to choose one item—as the speaker's intended item—from the contrast class of two non-jellyfish items on a screen. We would like to determine the moment that common ground considerations figure into the search for what the listener takes to be the intended item. We elaborate directly below.

To return to the experimental scenario, when the listener—who on past trials has associated the label *jellyfish* with one of three objects—processes the utterance ‘not the jellyfish’, it leaves her in a position to choose among two remaining ones. In the event that the jellyfish-like object is the only referent of three to have been named by the live confederate speaker, participants are obliged to choose randomly between the other two (this is the *one speaker–one precedent* condition). In the event that the live confederate speaker had also provided a name to a second item, for example, one that looks like a statue as ‘the statue’, we hypothesized that the participant would use the negative utterance to click on the one remaining unnamed object because listeners should prefer alternatives that are completely new (this is the *one speaker–two precedents condition*). These two conditions set up baselines for the critical test condition in which the live confederate speaker had, for example, coined *jellyfish* earlier while the other speaker (unbeknownst to the confederate) had provided a name to another of the two remaining items, ‘the statue’ in this case. This is the *two speakers–two precedents condition*.

The main question behind this research is the following: Do participants’ reactions in the critical two speakers–two precedents condition resemble those in the one speaker–one precedent condition or do they resemble those in the one speaker–two precedent condition? This could be further divided into two parts. Do participants’ response patterns in the critical two speakers–two precedents condition demonstrate that the listener immediately distinguishes the common ground established with the live confederate speaker from that established with the off-stage voice? The other concerns the time course of the eye-tracking data. Assuming that participants do take into consideration only the live confederate's perspective when she says ‘not the jellyfish’ (leading them to choose randomly between what are—to the confederate—two unnamed objects), do their search patterns resemble those found in the one speaker–one precedent condition or to those found in the one speaker–two precedents condition?

If participants’ responses and time course data in the two speakers–two precedents condition resemble those in the one speaker–one precedent condition, this would be direct support for the strong view [14,17]. This would indicate that participants are essentially ignoring the off-line exchange or are keeping track of their two conceptual pacts with high fidelity. On the other hand, if the participants in the critical two speakers–two precedents condition respond in a way that is similar to the one speaker–two precedents condition this would be strong support for the linguistic processing view. But this is not the only possible outcome that would lend support to the latter. Another possible outcome is that participants (i) do incorporate the two precedents linguistically (without considering the common ground of each) before they (ii) recognize downstream that the speaker could not have been aware that the listener has a name for one of the remaining items. In that case, the time course of participants should start off resembling those in the one speaker–two precedents condition and end up resembling those in the one speaker–one precedent condition. This would indicate that considerations of common ground do not emerge immediately.

### 1.3. The experiment

**Figure 1.**
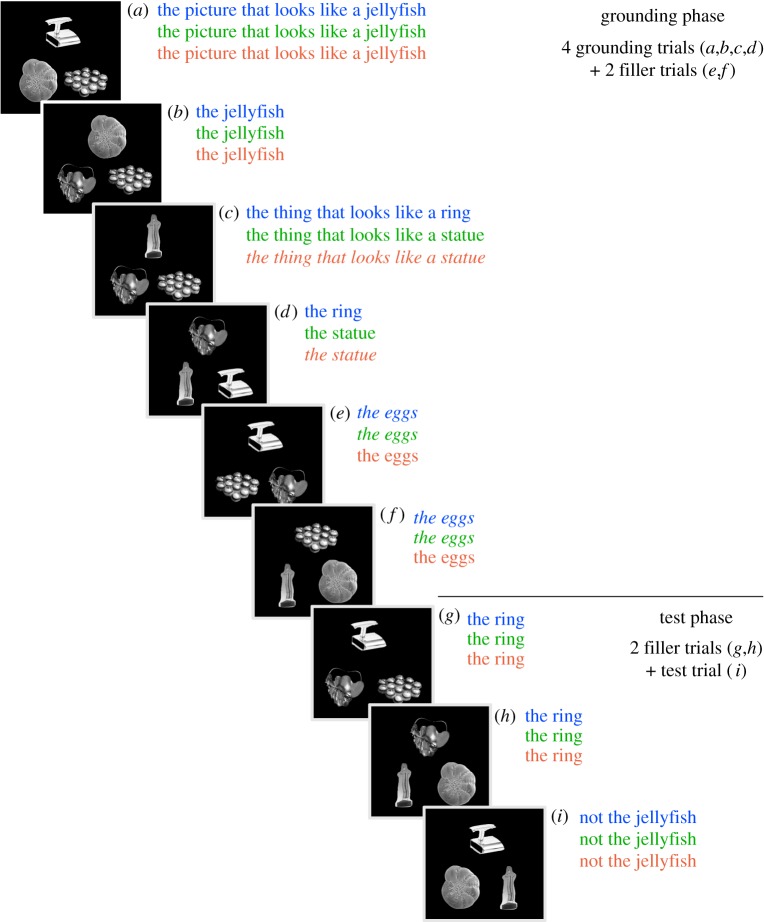
Schematic of a round. Each round consisted of nine trials (from *a*–*i*). Each trial consisted on one display with three objects and an instruction. One round was divided into a grounding phase with six trials (*a*–*f*) and a test phase with three trials (*g*–*i*). Next to the letter identifying each trial are the instructions given by the speakers for each condition: the first one, in blue, is the one speaker–one precedent condition; the second one, in green, is the one speaker–two precedents condition; the third one, in red, is the two speakers–two precedents condition. Instructions given by the remote speaker are italicized. The test trial is indicated by the letter ‘*i*’.

**Figure 2.**
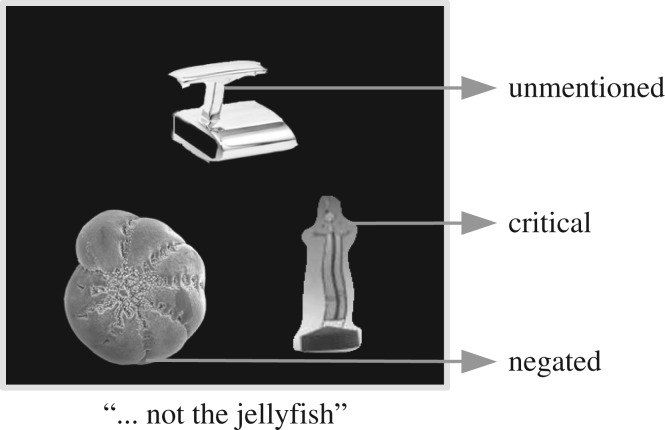
Example of test trial. The picture at the lower left side of the display is the negated alternative, which has been mentioned before twice by the live speaker in the grounding phase (trials *a* and *b*). The picture in the upper part is the unmentioned alternative, that has never been named. Finally, the picture on the lower right side is the critical alternative; what has happened to it in trials *c* and *d* during the grounding phase configured the different conditions. In the one speaker–one precedent was not named; in the one speaker–two precedents was named by the live speaker; and in the two speakers–two precedents was named only to the listener by the remote speaker through the headphones she was wearing.

As will become clear, the experiment was divided into a series of rounds, each consisting of two phases: a Grounding Phase that sets up precedents with certain speakers, followed by a Test Phase, which contained the test trial. We measured listeners’ eye movements and observed their referential choice as they listened to speech from the live speaker. All of the objects used in the displays were unusual and lacked conventional names. Figure 1 depicts a round and figure 2 is an example of a test trial.

The test trial provided the data to test our hypotheses. As can be seen in figure 2 (which corresponds to trial *i* in figure 1), a test trial included three alternative referents that varied with respect to their status in the discourse. The negated alternative (e.g. the jellyfish) was a named object that would be referred to through a negation. The unmentioned alternative is an object that had never been named by anyone in the discourse. Finally, there is the critical alternative, whose labelling varies across the three conditions. Whereas it is named previously by the confederate speaker in the one speaker–two precedents condition and named by the remote speaker in the two speakers–two precedents condition, it is not named at all in the one speaker–one precedent condition.

## 2. Material and methods

### 2.1. Participants

Twenty-four undergraduates (13 females and 11 males; mean *age*=22), who were all native speakers of Spanish, participated in exchange for 5000 Chilean Pesos (approx. 10 USD). Each participant received one of three stimulus lists created by rotating each item through all three conditions so that each participant saw a third of the items in each of the three conditions.

### 2.2. Design

The experiment had a one factor design with three conditions that differed as a function of the critical alternative. As is shown in figure 1, in the one speaker–one precedent condition, a filler object, not appearing on the test trial (*i*), was named in the grounding trials (*c* and *d*) by the live speaker as ‘the ring’, leaving the critical alternative unnamed; in the one speaker–two precedents condition, the live speaker named the critical alternative (the statue) in those same grounding trials; and in the two speakers–two precedents condition, the remote speaker (in italics) named the critical alternative, also in trials *c* and *d*.

### 2.3. Procedure

Upon arriving at the laboratory, the experimenter described the entire scenario and the task to both interlocutors before the actual roles of speaker (for the confederate) and listener (for the naive participant) were assigned. They were shown how the speaker and listener worked from different computer screens and that the two were prevented from viewing each other's screen. They were told that the task was for the listener to follow the instructions of the speaker on which object, out of two alternatives, to select. Participants were led to believe (and also, to believe that the confederate co-participant also believed) the following details about the procedure: (i) that on some trials the instructions would be given by a remote speaker; (ii) that these instructions were transmitted through headphones so that only the listener could hear them; (iii) that this remote speaker was a previous participant in the experiment whose instructions were recorded; and (iv) that while the listeners heard instructions from the remote speaker, the participant playing the role of speaker would see a blank screen and would not be able to hear the instruction. Up to this point, participants were also given the impression that the same two objects would appear on both screens.

**Figure 3.**
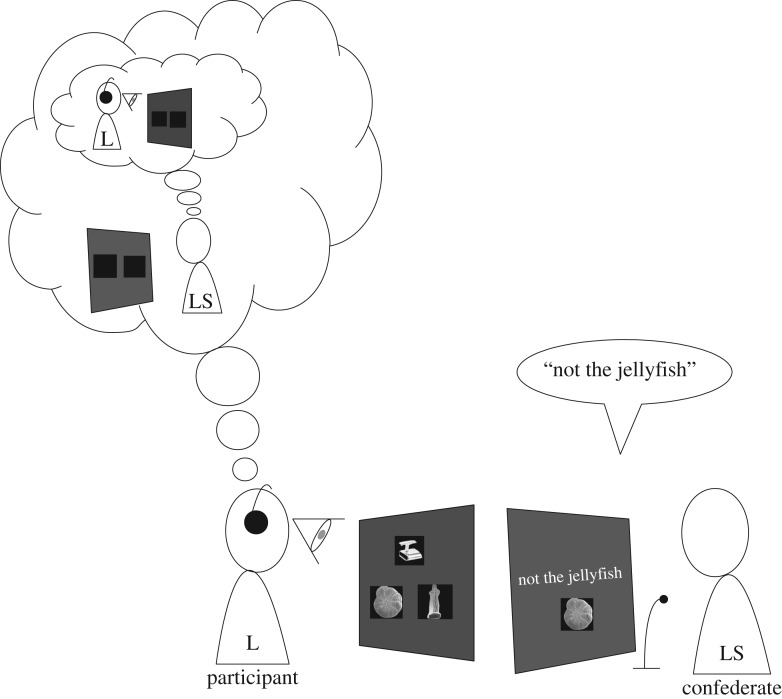
The experimental situation. The participant plays the role of listener (L) and a confederate the role of live speaker (LS). They cannot see each other's screen. On the listener's screen three objects appear; he believes that on the speaker's screen there are only two of these objects, not knowing which ones, and that the speaker believes that the same two pictures appear on his screen. In fact, the speaker is seeing the instructions she has to give. The listener is wearing headphones from which the instructions from the remote speaker are given only to him.

The next step was to assign the roles of speaker and listener to the participant and the confederate. In order to provide the impression of random assignment, the confederate and the participant drew slips of paper on which a single role was assigned. In fact, the drawing was rigged so that the participant would get the role of listener (both slips of paper were marked with the word ‘listener’) and the confederate announced that (s)he had drawn the role of speaker. Then, both sat in their places. The eye-tracking camera was placed in between the listener and his screen and, similarly, the microphone was placed between the confederate speaker and her screen through which she provided the instructions. Listeners wore headphones through which they heard—privately—the remote speaker instructions. Listeners selected (what they thought was) the intended object with the computer mouse. Figure 3 shows the experimental setting.

Before the task began, only the listener was told that he would actually see three alternatives, the two that appeared in the speaker's screen plus one more. Thus, listeners knew that speakers saw only two of the three alternatives, but did not know in advance which two. Importantly, the listener was led to believe that the speaker was unaware of any discrepancy between the displays. Now, the listener had another task—besides following the instructions from the speaker—which was to try to guess which was the object not appearing on the speaker's screen. With this additional feature of the cover story, we were able to generate a situation with referential ambiguity that allowed us to test our hypotheses: an instruction with a negation that rules out one alternative but leaves other two. Also, because the listener believed that the speaker was unaware of the discrepancy between their displays, there was no perception of uncooperative behaviour due to the ambiguity in the speaker's instructions. (Figure 3 depicts this situation.)

Figure 1 shows a schematic depiction of the task. For the sake of clarity, we will consider a single ‘trial’ an event in which the listener views a display and interprets a speaker's instruction to click on one of the objects. When we refer to a ‘round’, it is a collection of trials that includes the test trial, which was among the three last trials. The experiment had a total of 18 rounds, one for each experimental item.

The trials comprising a round can be subdivided into two ‘phases’: the grounding phase which consists of four trials that set up the names for the test trial objects (*a*,*b*,*c*,*d*) and two filler trials that serve to mask the real purpose of the grounding phase (*e*,*f*). The test phase contains the test trial (*i*) along with two filler trials (*g*,*h*). The trials were randomized with two restrictions: that the grounding phase always happened before the test phase, and that the trials in the grounding phase that introduced the name for an object (*a*,*c*,*e*), which usually had a heading of the sort ‘The picture that looks like a jellyfish’, always occurred before the trials that re-used that name without the heading (*b*,*d*,*f*). All behavioural measures (target selection and eye gaze data) were drawn from the test trials. In each test trial, the speaker used a negated referring expression, such as ‘not the jellyfish’ (‘no la medusa’ in Spanish), to indirectly identify the target.

As mentioned above, listeners saw three referential alternatives in the test trials: the *negated alternative*, the *unmentioned alternative* and the *critical alternative* (figure 2). The pictures associated with each of these alternatives had appeared with equal frequency in the trials preceding the test trial, with the unmentioned alternative seen but never named. Again, as outlined above, the three conditions were formed by either ignoring the critical alternative (much like the unmentioned alternative) or by manipulating who described the critical alternative (the confederate or the remote speaker).

In the two speakers–two precedents condition, the listener heard pre-recorded instructions while the confederate speaker presumably viewed a blank screen. To equate the number of pre-recorded instructions given to listeners across conditions, such instructions were given in the filler trials in the two other conditions (trials *e* and *f*).

### 2.4. Materials and apparatus

For each of the 18 experimental items, there were pictures of six different unconventional objects: one for the negated alternative, one for the unmentioned alternative, one for the critical alternative, plus three different filler objects. These were downloaded from the internet and were presented in greyscale so that they could not be identified using colour.

We tracked listeners’ eye movements using an EyeLink 1000 eyetracker (SR Research). The system used a remote tabletop camera, allowing relatively free head movement. Gaze data were recorded at a sampling rate of 500 Hz.

### 2.5. Data analysis

All analyses were performed and all the graphics created through R [31]. Our analysis considered both selection data (i.e. what the listener chose to click on) and eye-tracking data. For the selection data, we analysed the likelihood of choosing the unnamed alternative (a binary variable) using linear mixed-models with crossed random effects for subjects and items [32]. Because these data were categorical, we used a generalized linear mixed model with a binomial variance function and logit link. We tested all effects using model comparison, comparing models with identical random effects but with the fixed effect(s) of interest removed from one of the models. All models included the maximal random effects structure justified by the design [33]: by-subject and by-item random intercepts, as well as by-subject and by-item random slopes for each of the two variables used to code the three levels of the condition variable. These analyses were performed using the R add-on package lme4, v. 0.999999-2 [31].

Visual-world eye data are more difficult to analyse, because one needs to conduct a time-series analysis on a large dataset that accounts for temporal dependencies due to repeated sampling as well as for dependencies introduced by the experimental design. To overcome these challenges, we performed all analyses of the eye data using resampling techniques: specifically, bootstrapping to obtain confidence limits and an adapted version of the cluster randomization approach that is commonly used to analyse complex datasets in neuroimaging [34,35]. In adapting the approach for visual-world data, we followed Barr *et al.* [36]. Here, we provide an overview of the approach. Technical details are available in appendix A.

As our hypothesis concerned when certain effects emerge in our data, we wanted to use an approach that could localize events in time without the arbitrariness of the bin-by-bin analyses that are customarily used in visual-world research. In such analyses, there is usually no principled way to choose the size of each temporal bin in which effects are to be compared. Furthermore, researchers who use such analyses rarely correct for the additional familywise error introduced by conducting multiple comparisons. By contrast, the cluster randomization approach as applied to visual-world data avoids the problem of multiple comparisons, through the use of a single second-order test statistic known as a cluster mass statistic [34].

The basic logic is as follows. Multiple inferential tests are performed at each frame of eye-tracking data, and ‘clusters’ are identified as consecutive data frames in which all effects (i) are all significant at some specified alpha-level (usually 0.05), and (ii) are all in the same direction. In other words, any ‘run’ of consecutive frames that all show significant effects in the same direction constitutes a single ‘cluster’. The individual test statistics for each frame within a cluster are summed together to create a single second-order statistic for that cluster, the cluster mass statistic. The condition labels for the data are then shuffled according to some permutation logic and then the process is repeated on the transformed data. The permutation/analysis cycle is repeated a large number of times and the distribution of cluster mass statistics over the transformed data sets provides a null-hypothesis distribution for the cluster mass statistics on the clusters detected in the original data.

Because we were interested in generalizing over subjects and items, we performed separate cluster randomization analyses, one treating subjects as random factors and another treating items as random factors. Separate analyses were necessary because it is not clear within a resampling approach how to simultaneously generalize to both subject and item populations. We report the *p*-values for these analyses as *p*1 and *p*2 for subjects and items, respectively.

## 3. Results

### 3.1. Selection data

Table 1 shows the percentage of selections for each alternative in the three conditions. If one assumes that listeners incorporate common ground comprehensively (and that they use negations to search for alternatives), one ought to predict that participants will select the unmentioned alternative reliably in the one speaker–two precedents condition only; the two other conditions—the two speakers–two precedents condition and the one speaker–one precedent condition—ought to be similar and show that participants choose randomly from the two choices.

**Table 1.** Selection rate per alternative per condition.

	alternative		
condition	unmentioned (%)	critical (%)	negated (%)
one speaker–one precedent	50.0	49.3	0.7
one speaker–two precedents	80.6	16.0	3.5
two speakers–two precedents	56.2	43.1	0.7

The mixed-model analysis of the rate of unmentioned objects selection revealed a significant main effect of Condition (*χ*^2^ (2)=17.80, *p*=0.0001). Listeners selected the unmentioned alternative at a higher rate in the one speaker–two precedents condition (80.6%) than in the one speaker–one precedent (50.0%), (*χ*^2^ (1)=13.38, *p*=0.0003). The selection rate of the unmentioned object in the two speakers–two precedents condition (56.2%) was similar to that of the one speaker–one precedent condition (50.0%) (*χ*^2^ (1)=0.43, *p*=0.5104). Thus, at the end of the interpretation process, it appears that participants made their referential commitment by considering common ground information. That is, they realized that they need to consider that the critical object was named by the remote speaker in the two speakers–two precedents condition, so that when the live speaker says ‘not the jellyfish’, the listener is ultimately aware that speaker did not name the critical object.

### 3.2. Eye data

We now turn to the eye-tracking data in order to determine the extent to which we can isolate linguistic decoding processes in this task (i.e. independently of common ground considerations). We measure eye gaze to the three test-display objects at the offset of the negated expression (e.g. the end of the word ‘jellyfish’ in ‘not the jellyfish’). We used the offset because we assumed that listeners would begin by gazing at the negated object. If common ground is processed in parallel with the linguistic information in this task, gazing at the unnamed alternative in the two speakers–two precedents condition should resemble gazing at the unnamed alternative in the one speaker–one precedent condition because, in both cases, negated utterances such as ‘not the jellyfish’ leave open two possible reference assignments, including the unnamed alternative. On the other hand, if reference assignment is guided solely by the linguistic input (at least initially), gazing at the unmentioned alternative in the two speakers–two precedents condition ought to resemble gazing at the unmentioned alternative in the one speaker–two precedents condition because, in both cases, the listener has internalized a name for the two precedents (and without considering who assigned the names).

**Figure 4.**
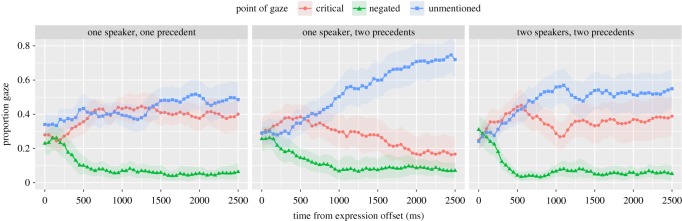
Proportion of time gazing at referential alternatives by condition. Points and lines are observed data; shaded regions represent 95% confidence intervals derived by bootstrapping subjects.

Figure 4 shows the proportion of time spent looking at each object for each condition between 0 and 2.5 s after the offset of the negated referring expression. As expected, listeners in the one speaker–one precedent condition show clear signs of being unable to decide between the two unnamed alternatives (the unmentioned and critical alternative). By contrast, in the one speaker–two precedents condition, listeners appear to look away from the negated alternative by about 200 ms and form a complement set consisting of the two remaining alternatives. From about 500 ms onward in the one speaker–two precedents condition, listeners show increased looks to the unmentioned alternative. These two conditions provide us with guideposts with which to appreciate gazing behaviour of the two speakers–two precedents condition.

The two speakers–two precedents condition initially resembles the one speaker–two precedents condition; indeed, these two conditions look remarkably similar until 1000 ms, after which looks to the unmentioned alternative seem to grow at a faster rate in the one speaker–two precedents condition. The overall pattern is consistent with an account in which eye gaze is guided, at least initially, by linguistic decoding.

These informal observations were supported by statistical analyses. We performed cluster randomization analyses on the log ratio of looks to the unmentioned versus the critical alternative because this pair of objects affords the clearest and most direct test of the hypotheses. The log ratio was derived from a baseline-category multinomial logit model fit to the data [37]. The log ratio is zero when there is equal preference for the two objects and is increasingly positive as preference for the unmentioned alternative increases. Likewise, if listeners prefer the critical alternative, the log ratio will become increasingly negative. Thus, to the extent listeners make common ground-based interpretations, the log ratio should be highest in the one speaker–two precedents condition.

**Figure 5.**
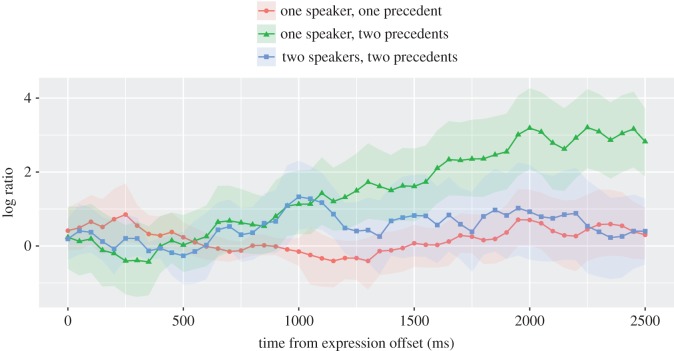
Log ratio by condition. Shaded regions represent 95% confidence intervals derived by bootstrapping subjects.

The log ratio data and confidence intervals are shown in figure 5. It is clear from this plot that between 500 and 1100 ms, preference for the unmentioned over the critical alternative diverged from the one speaker–one precedent condition and grew at similar rates regardless of whether the naming of the critical alternative was in common ground. A cluster randomization analysis comparing the average of the two speakers–two precedents and one speaker–two precedents conditions to the one speaker–one precedent condition detected a significant cluster (*p*1=0.004, *p*2<0.001) beginning at about 900 ms and continuing until the end of the analysis window (2500 ms). By contrast, the effect of common ground (difference between the one speaker–two precedents and the two speakers–two precedents conditions) did not emerge until about 500 ms later (*p*1=0.004, *p*2<0.001), starting at about 1600 ms and lasting until the end of the analysis window. This considerable delay between the onset of these two effects is consistent with the hypothesis that interpretation initially relies on the linguistic input before considerations of common ground come into play in the two speakers–two precedents condition.

In summary, early and momentary referential commitments, as judged by analyses of eye gaze, do not show the same pattern as late commitments and final referential assignments. Initial referential interpretation seems to be driven by the linguistic input while considerations raised by common ground emerge close to a half-second later. In this task, listeners appear to build upon the linguistic input in order to ultimately select the most pragmatically appropriate target.

## 4. General discussion

Reference resolution is a quintessential pragmatic phenomenon. As such, an utterance's linguistically encoded meaning ought to underdetermine its intended meaning and the output of linguistic decoding ought to interact with some form of common ground. The debate in the reference literature basically concerns the interaction. One dominating position has long been that common ground permeates the linguistic interpretation of an utterance from its earliest moments [7,8,14,17,21,24,25]. The other position is that, while intention reading is ultimately essential, it is not necessarily the directing force from the start. Linguistic decoding that does not fully take into account common ground can also be a source of interpretation, at least early on in sentence processing [10,16,18,23].

In this study, we tested the predictions from the two schools of thought as we aimed to determine the role and timing with which common ground comes into play when resolving reference in a scenario that has one or two precedents (established by up to two speakers). We introduced negation into the paradigm because it prompts a search for alternatives and we were interested in determining whether the listener's search would distinguish between alternatives based on the common ground with the speaker. By examining listeners’ selection choices, we could confirm that they ultimately do rely on common ground. Through eye-tracking measures, we found evidence indicating that participants’ initial interpretations are most likely driven by linguistic decoding, i.e. listeners do not rely on common ground in the early going.

How do these results fit into the literature on reference and common ground? As we indicated earlier, the traditional position proposes that common ground is the intrinsic context for comprehension [8]. It follows then that the current findings are inconsistent with this view as listeners’ eye-gazes suggest that referential search is independent of common ground up to 1000 ms after the offset of the referential utterance. By no means do we want to claim that common ground is not vital to comprehension. While we assume that listeners rely on common ground for comprehension, we only show that access to common ground is not immediate in the current, challenging paradigm.

These delayed effects of common ground also make our findings inconsistent with another approach that posits common ground is one of many cues combined immediately and simultaneously as part of a probabilistic constraint satisfaction process [14,17,21,24,25]. If common ground immediately constrained language processing on this task, the effect of common ground should have emerged no later than the earliest evidence for effects of referential processing. However, as we indicated earlier, there is no apparent difference between the one speaker–two precedents condition and the two speakers–two precedents condition until about 1000 ms, well after the gaze probability for the unmentioned alternative had already diverged from the one speaker–one precedent condition (about 500–1000 ms).

Our use of negation also makes a stronger case against constraint-based approaches than was possible in previous studies of referential precedents that used positive descriptions [16]. Unlike with non-negated expressions, effects of common ground do not have to fight against other effects of linguistic processing to be seen: the choice is guided entirely by the pragmatics of the situation. For instance, when a speaker re-uses an expression to refer to a target, common ground predicts that looks to the target should be faster and more when a conceptual pact exists with that speaker. However, when that description happens to also fit the target well independently of the previous discourse record, then looks to the target will be high anyway, making it more difficult to detect effects of common ground. For negated expressions, the semantics are used only to exclude an alternative from the set of possible referents, and thus the choice among the remaining set must be done on the basis of pragmatics alone, improving the chances for detecting effects of common ground.

The results are consistent with the idea that listeners begin interpreting referential utterance without the features of common ground (speaker-related labels) fully worked-out or registered. In this study's relatively complex task, we were able to isolate (the inevitable arrival of) common ground from linguistic processing. Until common ground considerations were taken up, the paradigm revealed that, for a full second, the listener's interpretation of ‘not the jellyfish’ in the two speakers–two precedents condition is processed as if the critical alternative has a name but without considering who had coined it. Indeed, participants in this task look away from objects that have a name, no matter who gave it.

Now we turn to several potential criticisms. The first concerns the use of negation, which is among the most difficult linguistic connectives to process. Psycholinguistic studies have shown how negations are effortful and a potential source of confusion [38]. The criticism is that perhaps a negative utterance in itself can generate a search for alternatives that is so overwhelming that it has the potential to undermine the attribution of common-ground related features. Our response to this potential critique is that any claims on the priority of negation-generating searches would only support the view that says that a linguistically generated process is a critical feature in this task, one that prevents common ground from gaining traction. Moreover, if negation had a general effect, the eye-tracking data from the one speaker–one precedent condition would look more similar to those in the other two, at least during the first 1000 ms. A related criticism is that perhaps this task underestimates effects of common ground, because the inferences required in this situation are unusually complex. This might seem especially compelling given claims from other research for immediate effects of common ground [17,24,26]. We cannot rule out this possibility, but also note that delayed effects of common ground also appear in the broken precedent case [19], where the inferences are much simpler. An important agenda for the field is to resolve whether these discrepancies in time-course reflect variation in the difficulty of inference, different levels of interactivity in the communicative situation [25] or methodological and analytical confounding [37].

A more global criticism concerns particular details of our experiment. Here, we focus on two features: (i) that we had a relatively small sample of 24 participants and (ii) that we used a speaker who was recorded. In response to the sample size, we point out that our results are rather consistent across subjects. For example, 19 out of 24 participants provide the same pattern in the way they ultimately rely on common ground to make their last referential commitment (see Results section). Additionally, it should be noted that there were multiple trials in each condition for each participant, and that the experiment used a within-participant design. The fact that common ground effects eventually appeared shows that it was adequately powered to detect such effects. We also point out that eye-tracking studies generally have a small number of participants, primarily due to the logistic demands that arise when running such experiments.

With respect to the other methodological concern (that we rely on one speaker who is recorded), we make three points. First, listeners do engage with the speaker in making common ground decisions, which is fully evidenced in their referential selections. Second, and more importantly, the main features of the task are generally natural and interactive with both speakers. For example, precedents are grounded collaboratively with the live speaker. And with the remote speaker's recorded instructions, they received audio feedback informing them whether their selection was correct or incorrect. Besides, the critical instruction, from which we collected the data, was always given by the live speaker. Finally, participants never questioned the idea that the second speaker was a real participant.

To summarize, this study examining the use of referential precedents in the processing of negated expressions revealed strong evidence that early interpretation processes are guided by linguistic considerations, and that effects of common ground are not immediate. Negated expressions provide a promising testing ground for theories of perspective taking in language comprehension by allowing greater isolation of pragmatic effects from linguistic effects. It is not the case that non-negated expressions are the best way to resolve controversies in the literature on perspective taking in language comprehension.

## Data accessibility

Data and scripts are available from the Dryad Digital Repository: http://dx.doi.org/10.5061/dryad.ct810 [39].

## Appendix A. Resampling approach for eye-data analysis

The cluster randomization approach is a popular solution in neuroimaging for analyses involving multiple tests at distinct locations and/or time points [34,35] (for prior adaptation of the solution to the analysis of visual world data, see [36]). This approach corrects for multiple comparisons by taking advantage of temporal correlations between subsequent observations. The logic is as follows. First, at each time point, a test is performed at a specified *α* level. Clusters are then identified by grouping together subsequent frames in which all effects are all significant and all are in the same direction. Once the clusters have been identified, a ‘cluster mass statistic’ is calculated for each cluster; usually the sum of all of the individual test statistics can be obtained by randomization (permutation tests). In particular, new versions of the dataset are created by shuffling condition labels according to some permutation scheme. Then, tests are performed on each of these newly created datasets as described above, and the maximum cluster mass statistic is identified. The vector of these statistics forms a null-hypothesis distribution for the original statistic.

We performed two cluster randomization analyses on the log ratio data, one to compare the average of the one speaker–two precedents and two speakers–two precedents conditions to one speaker–one precedent, and a second to compare the one speaker–two precedents and two speakers–two precedents conditions. The data were first divided into 50 ms time bins (25 samples of eye data). Log ratios were calculated for each bin by fitting a baseline-category multinomial logistic regression to the data [40], with the critical alternative as baseline. The log ratio of unmentioned to critical was the primary-dependent variable. The permutation scheme for the comparison to control was as follows. For each subject, we made a random decision whether to keep the original labelling for that subject, or whether to swap the condition labels. In the latter case, the condition labels for the three conditions would be swapped en masse for that subject; e.g. one speaker–two precedents would be replaced with two speakers–two precedents, two speakers–two precedents with one speaker–one precedent, and one speaker–one precedent with one speaker–two precedents. The permutation scheme for the comparison between one speaker–two precedents and two speakers–two precedents was similar, except the one speaker–one precedent condition labels remained fixed, and the decision for each subject was whether to keep or exchange the labels for the one speaker–two precedents and two speakers–two precedents conditions.

For each analysis, we created 9999 alternative versions of the dataset following the permutation scheme. For a given dataset, the *p*-values were determined by comparing the log ratio for that dataset to the distribution comprised by all other datasets. Each *p*-value was then converted into a test statistic *T* using the formula T=−log⁡(p) [36]; with this negative log transformation, the smaller the *p*-value, the larger the test statistic. All *T* values greater than −log⁡(0.05) were classified as significant, clusters were identified and a cluster mass statistic calculated for each cluster, which was the sum of all *T* values for that cluster.
